# Career coach preferences of medical students: coaching specialist or specialistic coach?

**DOI:** 10.1186/s12909-023-04882-1

**Published:** 2023-12-21

**Authors:** Daan A. H. Fris, Annelies E. M. van Vianen, Edwin A. J. van Hooft, Matthijs de Hoog, Anne P. J. de Pagter

**Affiliations:** 1 Department of Pediatrics, Erasmus Medical Center - Sophia Children’s Hospital, PO Box Sk-3284, Rotterdam, 3000 CA The Netherlands; 2 Work and Organizational Psychology, University of Amsterdam, PO Box 15919, Amsterdam, 1001 NK The Netherlands; 3 Department of Neonatal & Pediatric Intensive Care, Division of Pediatric Intensive Care, Erasmus Medical Center – Sophia Children’s Hospital, PO Box Sk-3284, Rotterdam, 3000 CA The Netherlands; 4 Department of Pediatrics, Willem-Alexander Children’s Hospital, Leiden University Medical Center, Leiden, The Netherlands

**Keywords:** Coach choice, Medical students, Career coaching, Social cognition, Warmth-competence framework

## Abstract

**Background:**

Medical students’ demand for career coaching is growing. However, little is known about what type of career coach they prefer. Using the Warmth-Competence Framework, we investigated if and why medical students prefer physician coaches compared to career psychologist coaches. We also examined whether students’ coach choice related to coaches’ amount of experience with medical students.

**Methods:**

In a two-by-two between participants vignette study (*n* = 147), we manipulated coach occupational background (physician vs. psychologist) and experience with coaching medical students (limited vs. considerable). Participants read one coach description, rated the likelihood that they would choose the coach, and rated the coach on dimensions of warmth and competence.

**Results:**

Students who evaluated a physician career coach were more likely to choose the coach than students who evaluated a psychologist career coach. Students expected that a physician career coach would better understand their situation and be better able to provide career information, while they expected a psychologist career coach to have better conversation skills, all of which were relevant to choosing a coach. Coaches’ experience with coaching medical students was unrelated to students’ coach choice and their assessment of the coach’s warmth and competence.

**Conclusions:**

Our findings highlight the relevance of coaches’ occupational background and have implications for the implementation of career coach interventions. Medical schools could help students choose a career coach by providing information about the coach qualities that students value. Future studies could investigate whether career coaches with different occupational backgrounds differ in coach behaviors and coaching effectiveness.

**Supplementary Information:**

The online version contains supplementary material available at 10.1186/s12909-023-04882-1.

## Introduction

Career coaching in medical education is gaining popularity in practice and research on the topic is growing [1–3]. Career coaching is a systematic and goal-oriented one-on-one intervention aimed to guide clients in their career [4, 5] that differs from other forms of individual guidance such as mentoring. While mentoring is a longer-term professional relationship focused on learning and professional development, career coaching is usually short-term and focused on specific career issues clients want to address (i.e., choices related to their future career). Unlike mentors, coaches are often recruited from outside clients’ organization [6] and do not necessarily have the same professional background as their clients [7]. Medical students have a particular need for career coaching in making career choices [8, 9], as they experience stress in the career decision-making process [10]. For successful coaching, a high-quality relationship between coach and client is critical [11]. Therefore, clients spend time choosing a coach with whom they expect to build a meaningful relationship and who can best support their coaching goals. This raises the question what coach characteristics medical students value when choosing a career coach.

Preliminary findings suggest that coaches’ educational and occupational background may affect students’ preference for a coach. That is, after their coaching trajectory medical students retrospectively mentioned that they found a coach with a background in medicine beneficial [12, 13]. However, additional research on students’ coach preferences is needed. First, the few studies describing medical students’ coach preferences [12, 13] asked students about their preferences only after they had completed their career coaching program. Second, extant research did not include a comparison between career coaches from different occupational backgrounds. Hence, we do not know whether medical students prefer a medically trained career coach more than, for example, a psychologically trained career coach. Third, if medical students would choose for a medically trained career coach, we do not know why they do so.

To address these research gaps, this study compares medical students’ preferences for a physician career coach (i.e., a physician trained in career coaching) with students’ preferences for a psychologist career coach (i.e., a psychologist with a master’s degree in career development and trained in career coaching) using an experimental vignette design. In addition to manipulating coaches’ occupational background (medicine vs. psychology), we manipulate coaches’ amount of experience in coaching medical students (considerable vs. limited experience). This allows us to examine whether students value coaches’ occupational background or their experience with medical students. Moreover, to understand the factors underlying students’ preferences for a specific coach, we draw upon the Warmth-Competence framework [14, 15] and assess students’ expectations of the coach’s warmth (e.g., understanding and trust) and competence (e.g., coaching skills, knowledge of medical careers).

The Warmth-Competence Framework (WCF) suggests that people assess other people on two dimensions: warmth and competence [14, 15]. Warmth refers to an assessment of ‘other’s perceived intent in the social context’ ([14], p. 63) and includes characteristics such as trustworthiness, morality, and understanding [16, 17]. Competence refers to ‘other’s ability to enact on intentions’ ([15], p. 77) and includes characteristics such as skills, confidence, and efficacy [14, 15]. In this study, we focus on three warmth characteristics: interpersonal trust, interpersonal safety, and understanding, and three competence characteristics: coaching skills, career information, and networking opportunities.

Interpersonal trust refers to positive expectations of the coach’s intentions [18, 19] and interpersonal safety refers to expectations of feeling safe to disclose personally meaningful information with the coach [19]. Interpersonal trust and safety foster an open and honest dialogue [20, 21], which is crucial for coaching success [20]. Clients likely prefer a coach who seems to have good intentions and to whom they dare to show their vulnerability. A third warmth-expectation relevant for coach choice is a coach’s understanding [22, 23]. Clients likely prefer coaches who understand them, as feeling understood promotes the quality of the client-coach relationship and client well-being [24–26].

Clients likely prefer a coach who is not only warm but also competent, that is, who has good coaching skills, provides career information, and helps them to explore career opportunities. Coaching skills refer to the coach’s ability to apply effective conversational techniques (e.g., feedback, challenging and solution-focused questions) [27, 28], which benefit a clients’ mood and progress [29]. Providing career information and career opportunities includes suggesting concrete career opportunities that suit the client and assisting in connecting with relevant professionals in the coach’s network. Gathering career information and exploring career opportunities are key for making career decisions [30, 31] as it informs clients about jobs that will match (or not match) their career preferences [32].

In summary, this study aims to answer the following questions: When choosing a career coach, do medical students prefer a physician career coach to a psychologist career coach? Can psychologists’ experience with coaching medical students compensate for a potential preference for physician career coaches? What coaching qualities do medical students associate with a physician career coach or a psychologist career coach? We examine these questions using the warmth-competence framework [14, 15]. This study contributes to theory and research on social cognitions and coaching by disentangling what cognitions underlie medical students’ expectations of a coach. In practice, the results of this study provide medical schools and career coaches with guidelines to optimize coaching programs, in particular, the choice of a coach.

## Methods

### Sample and design

The sample comprised preclinical medical students from a large medical school in the Netherlands. Students were in the third (and last) year of their bachelor’s. We used a 2-by-2 factorial between-participants design in which students were randomly assigned to one of four vignettes. The vignettes included a coach description with information on the career coach’s occupational background (physician vs. career psychologist) and experience with coaching medical students (considerable vs. limited experience; see Additional file 1).

###  Procedure^1^

This study was conducted in accordance with the data management and data protection regulations of the ethical review board (ERB) of the Faculty of Social and Behavioral Sciences of the University of Amsterdam. After ERB approval (IRB no. FMG-683), all third-year bachelor students (*N* = 461) received an e-mail invitation to participate in the study from the medical school’s study coordinator. Onto opening the survey link, students were presented an information letter including details on the study background, their right to withdraw, and the confidentiality of their provided data. After providing informed consent participants were directed to the survey.

Participants first answered questions about their demographics. Then, they were asked to imagine that they sought coaching to get support in making study and career choices, and that they were going to choose a career coach. After having read a description of a career coach (i.e., one of the four vignettes), they responded to questions about the probability that they would choose the coach they had just read about and their impression of this coach. Finally, students responded to questions about the described career coach (manipulation check). Upon completion, participants received a €7 gift card.

### Vignettes

A career coach was described in a single paragraph (see Additional file 1). Coach occupational background was manipulated by either describing a physician or a psychologist career coach (i.e., a talent development advisor). Coaches’ experience with coaching medical students was manipulated by the ratio of medical- versus other students that the coach had coached (85% medical students and 15% students from other programs, or vice versa). All vignettes contained similar information on the coach’s gender (all female to avoid varying implicit assumptions), training (yes), and general coaching experience (20 students per year in the last 5 years).

### Measures

Measures were based on existing scales, adapted to fit the context and purpose of the current study. Additional file 2 contains all items and response options. Table 1 displays the Cronbach’s alphas.

**Table 1:** Means, standard deviations, and correlation matrix for study variables

Variable	*α*	*M*	*SD*	1	2	3	4	5	6	7	8	9	10
1. Age	-	21.18	1.54	-									
2. Gender	-	1.72	0.45	-0.06	-								
3. Occupational background^a^	-	0.46	0.50	0.03	-0.07	-							
4. Experience with medical students^b^	-	0.54	0.50	-0.28**	-0.13	0.00	-						
5. Trust	.81	5.74	0.84	-0.14	0.14	-0.09	0.06	-					
6. Safety	.89	3.71	0.77	-0.04	0.11	-0.12	-0.04	0.53**	-				
7. Understanding	.73	3.34	0.91	-0.07	0.00	0.56**	0.02	0.13	0.08	-			
8. Coaching skills	.93	4.34	0.66	-0.17*	0.05	-0.36**	0.03	0.51**	0.41**	-0.18*	-		
9. Career information	.88	3.56	0.89	-0.06	0.01	0.25**	0.06	0.19*	0.11	0.62**	-0.02	-	
10. Networking possibilities	.90	3.40	1.02	-0.10	0.11	0.46**	0.06	0.06	0.09	0.60**	-0.12	0.52**	-
11. Coach choice	-	6.91	1.61	0.00	0.10	0.20*	0.08	0.13	0.17*	0.45**	0.12	0.49**	0.34**

^*^P < .05, **P < .01

^a^0 = career psychologist coach, 1 = physician coach

^b^0 = limited experience, 1 = considerable experience

*Coach choice* was assessed with a single question similar to previous research [33, 34], capturing the likelihood of choosing the coach (i.e., ‘How likely would it be for you to choose this coach?’), rated on a 10-point Likert scale (1 = very unlikely, 10 = very likely).

*Expected warmth of the coach* was assessed with three scales: interpersonal trust, interpersonal safety, and understanding. Interpersonal trust was measured with Qiu et al.’s [35] three-item scale, slightly adapted to fit our study context. We reworded the items to use identical scale anchors (1 = very unlikely to 7 = very likely) and we reversed one item. Interpersonal safety was measured with a three-item scale, based on the disclosure-based trust literature [36]. Understanding of the coach was measured with a three-item scale based on items by Lun et al. [24]. Items from the interpersonal safety and understanding scales were rated on 5-point Likert scales (1 = very unlikely, 5 = very likely).

*Expected competence of the coach* was assessed with three scales: coaching skills, career information, and networking possibilities. Coaching skills were measured with three items based on existing ability [37] and perceived competence scales [38]. Career information was assessed with three items, based on the lack of information about occupations scale [39]. Networking possibilities were assessed with three items, based on scales drawn from literature on mentoring and networking [40–42]. All items were rated on 5-point Likert scales ranging from 1 (very unlikely) to 5 (very likely).

### Manipulation check

We asked students about the coach’s vocation (physician, talent development advisor, attorney, or econometrician), bachelor’s study (medicine, psychology, law, or econometry), and the ratio medical versus other students that the coach has coached (85% medical students, 15% students from other master’s programs, or vice versa). When participants failed these manipulation checks, they were excluded from the analyses.

### Statistical analyses

Data were analyzed using MPlus 8.8. First, we analyzed the factor structure of the warmth and competence measures using Confirmatory Factor Analysis (CFA). The two CFAs supported the three-factor structure of the warmth and competence characteristics (see Additional file 3). Second, we used regression analysis to test the effect of occupational background, experience with medical students, and their interaction on coach choice. Third, we tested a path model, which included warmth and competence expectations as mediators to explain relations between the manipulated variables (and their interaction) and coach choice.

## Results

### Participants

In total, 153 students completed the survey (33.1%). The final sample size comprised 147 participants, as 6 students failed the manipulation check. The mean age was 21.2 years (*SD* = 1.54), and 71.4% of the participants was female (*n* = 105) which corresponds to the percentage of women in the student population. Table 1 shows the descriptive statistics.

### Main analysis

Table 2 presents the results of the regression analysis. Occupational background was significantly positively related to coach choice, indicating that students more likely chose a physician career coach (*M* = 7.27) compared to a psychologist career coach (*M* = 6.61). Experience with medical students and the interaction were both unrelated to coach choice, indicating that the coach’s experience with the target population is unimportant for coach choice and cannot compensate for the (psychologist) coach’s lack of a medical background.

**Table 2:** Results of path models predicting coach choice

Variables	Model 1												Model 2											
	Column 1			Column 2			Column 3			Column 4			Column 5			Column 6			Column 7			Column 8		
	Coach choice			Trust			Safety			Understanding			Coaching skills			Career information			Networking possibilities			Coach choice		
	*B*	*SE*	β	*B*	*SE*	β	*B*	*SE*	β	*B*	*SE*	β	*B*	*SE*	β	*B*	*SE*	β	*B*	*SE*	β	*B*	*SE*	β
Occupational background (1 = physician)	0.97*	0.38	.30	-0.27	0.24	-.16	-0.32	0.21	-.21	0.88**	0.19	.48	-0.61**	0.15	-.47	0.46*	0.21	.26	0.85**	0.23	.42	0.58	0.37	.18
Experience with medical students (1 = considerable)	0.54	0.35	.17	0.00	0.18	.00	-0.19	0.17	-.12	-0.10	0.20	-.05	-0.08	0.13	-.06	0.12	0.21	.06	0.05	0.22	.02	0.61	0.36	.19
Occupational background x experience with medical students	-0.58	0.52	-.15	0.22	0.29	.11	0.27	0.26	.15	0.28	0.25	.13	0.26	0.21	.17	-0.02	0.29	-.01	0.16	0.30	.07	-0.86	0.47	-.23
Trust																						-0.26	0.15	-.14
Safety																						0.23	0.16	.11
Understanding																						0.50*	0.22	.28
Coaching skills																						0.55**	0.20	.22
Career information																						0.57**	0.19	.32
Networking possibilities																						0.03	0.15	.02
R^2^			.06			.02			.02			.33			.14			07			.21			.34

Unstandardized estimates (*B*), standard errors (*SE*), standardized coefficients (β) are presented. Column 1 reports results of Model 1, including direct relations between the manipulated variables and their interaction and coach choice. Column 2 until 7 report results of Model 2 including the same relations as Model 1 plus relations between the manipulated variables and their interaction with coach expectations, and relations between coach expectations and coach choice. Both models yielded a perfect fit with the data (Model 1: χ2/df = N/A, *p* < .001, SRMR = .00, CFI = 1, RMSEA = .00; Model 2: χ2/df = N/A, *p* < .001, SRMR = .00, CFI = 1, RMSEA = .00)

^*^P < .05, **P < .01

**Fig. 1:**
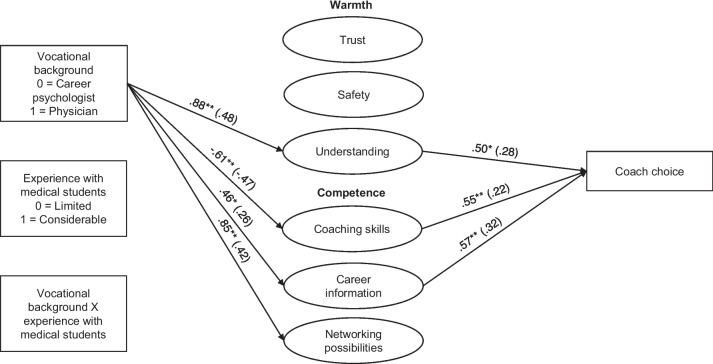
Results of mediation path model Note: *p* < .05, ** *p* < .01. Unstandardized estimates are reported, with standardized coefficients in brackets. Only significant paths are presented. Paths from occupational background, experience with medical students, and the interaction term to warmth- and competence expectations as well as to coach choice were modeled. All paths from the warmth- competence expectations to coach were modeled as well

To examine students’ expectations underlying their coach choice, we tested a path model including students’ warmth and competence expectations about the coach as mediators (see Tables 2 and 3 and Fig. 1). Paths were added from occupational background, experience with medical students, and their interaction to the warmth (i.e., interpersonal trust, interpersonal safety, and understanding) and competence (i.e., coaching skills, career information, and networking opportunities) expectations as well as coach choice. Also, paths from students’ warmth and competence expectations to coach choice were added. The expectations were allowed to correlate. We assessed indirect effects from the manipulated variables to coach choice through expectations about the coach using bias-corrected bootstrapping confidence intervals (5000 samples) [43].

**Table 3:** Estimates and confidence intervals of total, direct, and indirect effects for coach choice

Path	Unstandardized			Standardized		
	Lower 2.5%	Point estimate	Upper 2.5%	Lower 2.5%	Point estimate	Upper 2.5%
Total effects from occupational background to coach choice	0.20	0.97	1.76	.07	.30	.52
Total indirect effects	-0.12	0.38	0.95	-.04	.12	.28
Specific indirect effects						
Occupational background ➔ trust ➔ coach choice	-0.03	0.07	0.32	-.01	.02	.10
Occupational background ➔ safety ➔ coach choice	-0.29	-0.08	0.02	-.09	-.02	.01
Occupational background ➔ understanding ➔ coach choice	0.10	0.43	0.97	.03	.14	.30
Occupational background ➔ coaching skills ➔ coach choice	-0.70	-0.34	-0.10	-.21	-.10	-.03
Occupational background ➔ career information ➔ coach choice	0.04	0.26	0.66	.01	.08	.20
Occupational background ➔ networking possibilities ➔ coach choice	-0.23	0.02	0.31	-.07	.01	.10
Direct effect of occupational background to coach choice	-0.10	0.58	1.33	-.04	.18	.40

Bias corrected bootstrapping confidence intervals are reported

Regarding the warmth expectations, coach’s occupational background (0 = career psychologist, 1 = physician) was significantly positively related to understanding, but not significantly related to interpersonal trust and interpersonal safety. Thus, students expect a physician career coach to better understand the situation they are in regarding their career choices. Regarding the competence expectations, occupational background was significantly negatively related to coaching skills, and positively to career information and networking possibilities. Thus, students expect a psychologist career coach to have better coaching skills, while they expect a physician career coach to offer more career information and networking opportunities. Experience with medical students and the interaction term were unrelated to students’ expectations about the coach, indicating that coach’s experience with medical students does not influence students’ warmth and competence expectations about the coach and does not buffer the effects of coaches’ occupational background.

Students’ expectations of coach understanding positively related to coach choice, while expectations of coach interpersonal trust and safety were unrelated to coach choice. Furthermore, expectations of coach coaching skills and career information positively related to coach choice, whereas expectations of coach networking possibilities were unrelated to coach choice.

Last, we assessed indirect effects from coach occupational background to coach choice through students’ expectations of coach warmth- and competence (see Table 3). We found significant positive indirect effects through expectations of the coach’s understanding and career information. The indirect effect of occupational background on coach choice through expectations of the coach’s coaching skills however, was significant and negative. Additional indirect effects through expectations of trust, safety, and networking possibilities were non-significant. The direct effect of coach occupational background on coach choice became non-significant when the mediators were added to the model, suggesting full mediation.

## Discussion

This study aimed to (a) provide insight into medical students’ career coach preferences regarding coaches’ occupational background, and (b) explore the mechanisms through which career coaches’ occupational background explains medical students’ coach choice. Using an experimental between-participants vignette design, we compared medical students’ preferences for a physician career coach and a psychologist career coach. Our findings indicate that medical students prefer a physician career coach to a psychologist career coach and that a coach’s experience with coaching medical students does not weaken this preference. Students expect a physician career coach to understand them better and be better able to provide relevant career information compared to a psychologist career coach, which they take into account in their coach choice. Students do see advantages in a psychologist career coach because they expect this coach to have better coaching skills, which they factor in when choosing a coach.

### Findings and implications

This study demonstrates that medical students prefer a medically trained coach to a psychologist coach pre-coaching. This finding extends prior research that suggested that medical students found a medically trained coach beneficial post-coaching [12, 13], and corresponds with studies demonstrating that clients prefer a coach with a similar occupational background [44, 45]. A coach’s experience with medical students did not influence coach choice and could not compensate for a background in psychology.

Regarding warmth characteristics, a physician career coach as compared to a psychologist career coach evoked in students the expectation that the coach would understand them, irrespective of the coach’s experience with coaching medical students. Apparently, coaches are only perceived as better able to understand students when they have been through medical school themselves. This could be related to the strong professional identity and in particular the sense of social exclusivity that medical students develop during their studies [46]. Perceptions of exclusivity can lead medical students to expect that only people with a medical background and not others (e.g., psychologists) will understand the context in which they study or work. This expectation, in turn, influences students’ coach choice. In contrast to the effects on understanding, a coach’s professional background did not affect students’ expectations of trust and interpersonal safety. Medical students’ similarity to a physician coach along with their expectation that a career psychologist is better qualified may have resulted in a null effect on the expectation that the coach can be trusted. However, students’ expectations of trust and safety were not associated with their coach choice.

Regarding competence characteristics, students expected physician career coaches to have poorer coaching skills than psychologist career coaches. Since all vignettes described that the coach had professional coaching training, this suggests that medical students expect the occupational background of the psychologist to be of additional value to their coaching skills. Students seem to value advanced coaching skills because higher perceived coaching skill was related to coach choice. In contrast, students expected that a physician career coach compared to a psychologist career coach was better at providing career information and networking opportunities. A coach’s experience with coaching medical students did not affect these expectations. Students seem to assume that coaches need a medical background for providing relevant career information and networking opportunities to clients.

### Implications for medical education

Findings of this study have implications for the implementation of coaching programs in medical education. Because physician career coaches with professional coach training are scarce, medical schools involve psychologist career coaches in coaching programs. To make student participation in coaching by a career psychologist more attractive, students’ existing ideas about these coaches need to be challenged. For example, students may be told that these coaches are knowledgeable about broadening career opportunities within the medical field. Similarly, medical schools could also challenge students’ ideas about the poor coaching skills of physician career coaches, for example, by emphasizing these coaches’ training in conversational skills. Challenging students’ beliefs about coaches and emphasizing their specific qualities may support students in their coach choices.

Additionally, medical schools could inform students about the goals of coaching and the methods used. Although career information is important in making career decisions and career coaches can share information [30, 31], informing is not the focal aim of coaching [47]. Rather, coaching focuses on self-directed change [47]. Career coaches can stimulate students to reflect on and gain insight in their prior career experiences (i.e., engage in self-exploration) [30, 48]. In addition, career coaches can facilitate students in learning skills to make career decisions. The value of these decision-making skills extends beyond the coaching trajectory because these skills will be useful throughout their careers.

### Limitations and suggestions for future research

The experimental vignette design of the current study allows us to draw strong conclusions about the influence of a coach’s occupational background on students’ expectations and coach choice. Moreover, the study’s procedure ensured spontaneous coach assessments which were not affected by their responses to the warmth and competence measures. Still, some limitations should be taken into account when interpreting our findings.

First, future research is needed to examine the generalizability of our findings using different designs, settings, and samples. This study served as an initial exploration of how coach’s occupational background relates to medical student coach choices. Our between-participants design fitted this aim well, as students could not compare coach descriptions therefore remaining unaware of the manipulation. However, students participating in coaching programs will usually be able to base their choice on a comparison between different coaches. Therefore, a next research step is to use a within-participants design (i.e., where students assess multiple vignettes). Also, participants in the current study were instructed to imagine that they were seeking coaching. Therefore, the current sample may not fully mirror the preferences of students who actively seek coaching. Such students might be more motivated to make a coach choice and better informed about the goals and methods of coaching. This could influence their coach preferences. To minimize this potential bias, we provided a brief introduction to coaching to ensure that all participants had a basic understanding. Future research could investigate medical students’ actual coach choices.

Next to investigating the ecological validity of our findings, future research could compare coach preferences across different populations. For example, coach preferences of practicing physicians who seek coaching to improve their work-family balance may differ from preferences of students seeking career support. Besides, future studies could compare the effectiveness of physician and career psychologist’ career support for medical students, for example in reducing career decision-making stress [10]. Students’ assessment of the warmth and competence of the coach could be measured post-coaching as to see whether students’ pre-coaching expectations hold after their trajectory. Finally, if psychologist career coaches prove to be equally or more effective in coaching medical students in their career decision making, future research could investigate factors that alleviate medical students’ preference for physician career coaches. For example, positive testimonials may be used to influence coach choices.

### Conclusion

In conclusion, medical students prefer physician career coaches to psychologist career coaches, as they expect physicians to better understand them and better able to provide career information. However, psychologist career coaches were expected to have better coaching skills, which students consider when choosing a career coach. Emphasizing the coach characteristics that students deem important could benefit the implementation of career coaching interventions. Future research should investigate the effectiveness of coaches with varying occupational backgrounds in supporting students’ career decision-making process.

### Supplementary Information

**Additional file 1.** Example vignettes.

**Additional file 2.** Measures.

**Additional file 3.** Description of CFA and corresponding tables.

## Data Availability

Data will be publicly shared upon acceptance. Data can be requested through contacting the corresponding author. The vignettes and scale items are included in the additional files.

## Abbreviations

WCF: Warmth- Competence Framework
CFA: Confirmatory Factor Analysis

## Footnotes

1 Anonymized data are publicly available in OSF through the following link: https://osf.io/62f3d/.
