# Supplementary material for: Career coach preferences of medical students: coaching specialist or specialistic coach?
Source: BMC Med Educ. 2023 Dec 21;23:988. doi: 10.1186/s12909-023-04882-1 (PMC10740245; doi:10.1186/s12909-023-04882-1)
Supplement: Supplementary file 1 — Additional file 1. Example vignettes. [file 12909_2023_4882_MOESM1_ESM.docx]

Additional File 1 - Example vignettes

*Physician coach, considerable experience*

The coach is a woman and works as a physician. After completing her Bachelor's degree in Medicine, she also pursued a Master's degree in Medicine. In addition to her work as a physician, she completed a coaching training program and also coaches students. Over the past 5 years, she coached approximately 20 students per year in making career choices. Approximately 85% of these students were medical students, while the remaining 15% pursued degrees in various other fields.

*Physician coach, limited experience*

The coach is a woman and works as a physician. After completing her Bachelor's degree in Medicine, she also pursued a Master's degree in Medicine. In addition to her work as a physician, she completed a coaching training program and also coaches students. Over the past 5 years, she coached approximately 20 students per year in making career choices. Approximately 15% of these students were medical students, while the remaining 85% pursued degrees in various other fields.

*Career psychologist coach, considerable experience*

The coach is a woman and works as a talent development advisor. After completing her Bachelor's degree in Psychology, she pursued a Master's degree in Career Development. In addition to her work as a talent development advisor, she completed a coaching training program and also coaches students. Over the past 5 years, she coached approximately 20 students per year in making career choices. Approximately 85% of these students were medical students, while the remaining 15% pursued degrees in various other fields.

*Career psychologist coach, limited experience*

The coach is a woman and works as a talent development advisor. After completing her Bachelor's degree in Psychology, she pursued a Master's degree in Career Development. In addition to her work as a talent development advisor, she completed a coaching training program and also coaches students. Over the past 5 years, she coached approximately 20 students per year in making career choices. Approximately 15% of these students were medical students, while the remaining 85% pursued degrees in various other fields.
