# Supplementary material for: Career coach preferences of medical students: coaching specialist or specialistic coach?
Source: BMC Med Educ. 2023 Dec 21;23:988. doi: 10.1186/s12909-023-04882-1 (PMC10740245; doi:10.1186/s12909-023-04882-1)
Supplement: Supplementary file 2 — Additional file 2. Measures. [file 12909_2023_4882_MOESM2_ESM.docx]

Additional File 2 – Measures

Coach choice^1^

1. How likely would it be for you to choose this coach?

Trust^2^:

1. This coach can be trusted.
2. This coach is reliable.
3. This coach is someone to be distrusted. (R)

Safety^3^:

1. With this coach, I feel comfortable sharing information, feelings, and thoughts.
2. With this coach, I feel safe to share personally meaningful information.
3. I can show my true feelings and emotions to this coach.

Understanding^3^:

1. This coach can understand what it is like for me to choose a career path.
2. This coach can understand the obstacles and difficulties I face in making a career choice.
3. This coach can try her best but will never truly understand what it is like for me to make a career choice. (R)

Coaching skills^3^:

1. This coach has good coaching skills (e.g. listening skills, conversation techniques).
2. This coach possesses the right skills to coach.
3. This coach is an expert in her work as a coach.

Career information^3^:

1. This coach can provide me with information about a variety of career options.
2. This coach can give me specific information about different career options (e.g. information about development opportunities, information about further education).
3. This coach can inform me about career opportunities that interest me.

Networking possibilities^3^:

1. This coach can connect me with (professional) contacts who can provide me with information about career paths and options.
2. This coach has a network of contacts or friends that can help me increase my career opportunities.
3. This coach has a network that I can rely on for support in my career.

^1^Response scale: 1-10 (very unlikely – very likely)

^2^Response scale: 1-7 (very unlikely – very likely)

^3^Response scale: 1-5 (very unlikely – very likely)
