# Supplementary material for: Career coach preferences of medical students: coaching specialist or specialistic coach?
Source: BMC Med Educ. 2023 Dec 21;23:988. doi: 10.1186/s12909-023-04882-1 (PMC10740245; doi:10.1186/s12909-023-04882-1)
Supplement: Supplementary file 3 — Additional file 3. Description of CFA and corresponding tables. [file 12909_2023_4882_MOESM3_ESM.docx]

Additional File 3 – Description of CFA and corresponding tables

We tested the factor structure of warmth (i.e., expected interpersonal trust, expected interpersonal safety, and expected understanding) and competence (i.e., expected coaching skills, expected career information, and expected networking opportunities) expectations separately (see Table S3A and S3B, respectively). For both warmth and competence, we compared a three-factor model (i.e., a model in which the three expectations loaded on their respective factors) with a common factor model (i.e., a model in which all three expectations loaded on the same warmth or competence factor). We followed Hu and Bentler’s (1998) recommendations for model fit assessment using SRMR and RMSEA values, and complemented these with CFI values. A good fit is indicated by SRMR values of .08 (or lower), RMSEA values of .06 (or lower), CFI values of .95 (or higher) (Hu & Bentler, 1998).

For both the warmth and competence expectations, the three-factor model fitted well (χ2/df = 1.74, *p* = .01, SRMR = .05, CFI = .97, RMSEA = .07, χ2/df = 37.79, *p* = .04, SRMR = .04, CFI = .99, RMSEA = .06, respectively) and better than the common factor models (Δχ2(3) = 249.19, p < .001, Δχ2(3) = 537.85, *p* < .001, respectively). Therefore, we adhered to the three-factor structure in subsequent analyses.

Table S3A

*Confirmatory Factor Analyses of Warmth Expectations*

| Model | χ2 | *df* | χ2/d*f* | *p* | SRMR | CFI | RMSEA | Model comparison | Δχ2 | Δd*f* | *p* |
| --- | --- | --- | --- | --- | --- | --- | --- | --- | --- | --- | --- |
| 3-factor model | 41.69 | 24 | 1.74 | .01 | 0.05 | 0.97 | 0.07 |  |  |  |  |
| 1-factor model | 290.89 | 27 | 10.77 | <.001 | 0.15 | .59 | 0.26 | 3-factor model > 1-factor model | 249.19 | 3 | <.001 |

Table S3B

*Confirmatory Factor Analyses of Competence Expectations.*

| Model | χ2 | *df* | χ2/d*f* | *p* | SRMR | CFI | RMSEA | Model comparison | Δχ2 | Δd*f* | *p* |
| --- | --- | --- | --- | --- | --- | --- | --- | --- | --- | --- | --- |
| 3-factor model | 37.79 | 24 | 1.58 | .04 | 0.04 | 0.99 | 0.06 |  |  |  |  |
| 1-factor model | 575.65 | 27 | 21.32 | <.001 | 0.22 | .42 | 0.37 | 3-factor model > 1-factor model | 537.85 | 3 | <.001 |
